# Functional and Radiological Outcome in Atypical Subtrochanteric Femur Fracture After Surgical Fixation: A Retrospective Observational Study

**DOI:** 10.7759/cureus.29201

**Published:** 2022-09-15

**Authors:** Debashish Mishra, Tanmoy Mohanty, Saurav N Nanda, Ankit Gulia, Srikant Konchada, Saswat Samant, Ashok Gachhayat, Divyadeep Goyal, Bodanapu Sandeep, Swatantra A Mohanty

**Affiliations:** 1 Orthopaedics, Kalinga Institute of Medical Sciences, Bhubaneswar, IND; 2 Orthopaedics and Traumatology, Kalinga Institute of Medical Sciences, Bhubaneswar, IND; 3 Orthopaedics, Apollo Hospital, Bhubaneswar, IND

**Keywords:** subtrochanteric, fracture healing, osteoporosis, bisphosphonates, atypical femur fractures

## Abstract

Background: Treatment for osteoporosis can have catastrophic side effects, including the uncommon fracture known as an atypical femur fracture (AFF), which is related to the long-term usage of antiresorptive agents. Bisphosphonate therapy may lead to significant and chronic suppression of bone turnover, impairing the bone's remodelling property and finally leading to incomplete or complete atypical femur fracture. AFF was defined by the American Society for Bone and Mineral Research (ASBMR) Task Force in 2010 and is far less prevalent than proximal femur (hip) fracture, with an incidence of 2 to 78 per 100,000 patients per year following two to eight years of bisphosphonate therapy, respectively. Due to the rarity of the fracture, it is still not clear what the functional and radiological outcome will be after surgery.

Aim: To identify the functional and radiological outcomes of surgical fixation of atypical femur fractures.

Methods: The study was conducted in a tertiary healthcare centre after scientific and ethical clearance from the competent authority. Between January 2018 and December 2021, individuals who were diagnosed with an atypical subtrochanteric femoral fracture associated with the use of bisphosphonates and treated surgically were retrospectively evaluated. The study's inclusion and exclusion criteria were used to include 20 patients. The features of an atypical subtrochanteric fracture were congruent with the radiographic findings. Most of the patients were treated with internal fixation with intramedullary osteosynthesis in standard with or without plate osteosynthesis. They were then followed up for a year to look at the functional and radiological outcomes.

Results: All of the 20 patients who were included had an atypical subtrochanteric fracture, with 15 of them being female and 5 of them being male. The patients' mean age at surgery was 65.12 (range 49 to 82) years, and their average history of bisphosphonate use was 3 (range 2.5 to 5) years. All patients were treated surgically. We found that five months was the mean period for bone union (p = 0.990). Within six months, bone union was achieved in 11 patients (55 %) (p = 0.884). Five patients (about 25%) had implant failure and non-union, requiring two to three revision surgeries. At three, six, and nine months, the mean visual analogue score (VAS) was 4.14, 3.12, and 1.85, respectively. The modified Hip Harris Score had a mean of 72.66 and 15 patients (about 75% of them) could walk normally again after a fracture. The mean of the modified HHS was 72.66, and the VAS at three, six, and nine months was 4.14, 3.12, and 1.85, respectively.

Conclusion: AFFs are rare fractures that must be treated effectively, and most of them require surgery. Successful treatment of AFF is possible by the use of intramedullary fixation, which enhances axial stability, serves as an internal splint, and lessens the likelihood of implant failure. A good functional and radiological prognosis can come from a stable fixation and a fracture that has been reduced anatomically.

## Introduction

The term "atypical femoral fracture" (AFF) refers to a distinct kind of stress or insufficiency fracture that differs from conventional femoral fractures in a wide range of clinical aspects [1,2]. In 2010, the American Society for Bone and Mineral Research (ASBMR) constituted a task force and published a case definition for AFFs [3], and in 2014, a revised AFFs case definition was published [4]. First, the fracture line can be found anywhere along the bone between the lesser trochanter and the femoral supracondylar flare. Second, the injury is the result of a fall from a standing height or less, or there is no or minor trauma history. The radiographic findings are also distinctive, including a transverse or short oblique fracture pattern, a lack of comminution with a medial spike, and periosteal or endosteal thickening of the lateral cortex at the fracture site. Additionally, minor characteristics of AFFs were defined, but they lacked relevance to the case definition [1].

In the United States, there are 20 to 30 subtrochanteric femur fractures and femoral shaft fractures for every 100,000 person-years [5,6]. It has been estimated that, per 100,000 people per year, the incidence of atypical fractures ranges from 0.9% to 78.2% [7,8]. These figures are based on a study of radiography studies [7,8]. The ASBMR task committee [3] found that the number of cases went from 2 per 100,000 patients per year after two years of bisphosphonate therapy to 78 per 100,000 patients per year after eight years. In research by Dell et al. [4], it was found that 53.9% of patients who continued to use bisphosphonates three years or more after their primary atypical femoral fracture experienced an atypical femoral fracture on the opposite side. The severity of pain, fracture pattern, the presence of other injuries, and the patient's overall health all play a role in how atypical femoral fractures are treated. Atypical femoral fractures that are incomplete but do not cause symptoms can typically be treated conservatively. However, fractures that are complete or partial but cause pain must be treated surgically [9]. The functional and radiological outcome after surgical fixation is still unclear from existing literature due to the fracture's rarity. Our study's goal is to determine the functional and radiological outcomes of AFF surgical fixation.

## Materials and methods

The study was conducted in a tertiary healthcare centre and has been authorised by the scientific and ethical committees with IRB approval number KIITS/005/OR2022. Patients diagnosed with atypical subtrochanteric femoral fractures between 2018 and 2021 were retrospectively reviewed. All of the clinical presentations, comorbidities, chronic drug use records, radiological features, surgical techniques, outcome measures, and follow-up data were written down.

After applying strict inclusion and exclusion criteria, we enlisted 20 patients in the research group (Table 1).

**Table 1:** The patient's demographic characteristics. The data are displayed as the mean (median). F/M: female/male, ALP: alkaline phosphatase.

Atypical fracture	All (mean)
Age (mean-years)	65
Sex (F/M)	15/5
H/o bisphosphonates (year)	3
Serum calcium (mg/dl)	7.928
Serum phosphorus (mg/dl)	3.7
Vitamin D3 (ng/mL)	15.88

Patients who were on bisphosphonate therapy and presented with an atypical femur fracture were included in the study. Patients who had concurrent bilateral fractures, open wounds, cancer, or were being treated conservatively were excluded from the research. All cases included had a mean age of 65 years (49-82 years). The mean bisphosphonate treatment duration was three years (2.5-05 years).

The fracture site was opened in all the cases to achieve anatomical reduction, followed by intramedullary nailing in the standard manner. In the case of suspicion of stability, plate augmentation was done along with the nailing. All the patients were mobilised with toe touch on the first post-operative day. Patients were allowed for full weight-bearing mobilisation six weeks after surgery. All the patients were followed up for at least one year from the date of surgery.

In order to get a radiological assessment of the patient's condition after surgery, follow-up radiographs were taken at 1, 3, 6, and 12 months. Two independent observers assessed a variety of radiological outcomes, including fracture union, delayed union, and implant material failure.

Independent reviewers prospectively performed functional assessments using the modified Harris Hip Score (HHS) and visual analogue scale at three months, six months, and one year after surgery. At the one-year post-operative follow-up, walking capacity was also tested, and patients were divided accordingly. Pain, daily activities, mobility, and range of motion can be assessed functionally using a modified HHS score [10]. The highest possible score is 100 points. The NRS pain score, also called a VAS score, was used to figure out how severe the patients' reported pain was. This score ranges from 0 (no pain) to 10 (the worst pain possible).

## Results

**Figure 1:**
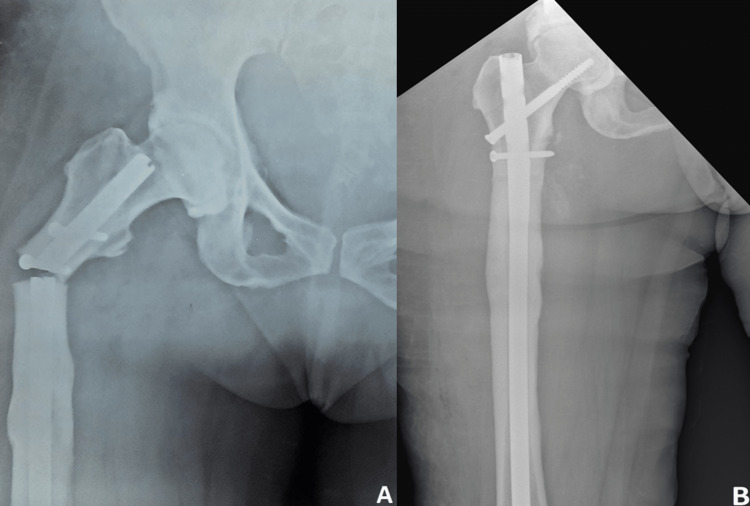
Pre-operative (A) and post-operative (B) one year follow up radiograph of implant failure.

All of the 20 patients who were included had an atypical subtrochanteric fracture, with 15 of them being female and 5 of them being male. The patients' mean age at surgery was 65.12 (range 49 to 82) years, and their average history of bisphosphonate use was 3 (range 2.5 to 5) years. A proximal femoral nail with or without plate osteosynthesis was used to treat 10 patients; an intramedullary interlocking nail was used to treat 8 patients; and the remaining 2 underwent long plate fixation. These patients experienced no significant intraoperative problems. All patients had good wound healing post-op. We found that five months was the mean period for bone union (p = 0.990). Within six months, bone union was achieved in 11 patients (55 %) (p = 0.884). Five patients (about 25%) had implant failure (Figure 1) and non-union, requiring revision surgeries (Table 2).

**Table 2:** Numerical rating scale. The data are displayed as the mean (median).

Numerical rating scale (VAS score)	All (mean)
Post-operative	7.17
Post-operative three months	4.14
Post-operative six months	3.12
Post-operative nine months	1.85
Post-operative one year	1.11
Recovery to pre-OP walking ability (%)	15 (75%)

At three, six, and nine months, the mean visual analogue score (VAS) was 4.14, 3.12, and 1.85, respectively (Table 3).

**Table 3:** Clinical outcomes.

Clinical outcomes	All
Time to union (mean - months)	5
Six months union (%)	55%
Nine months union (%)	75%
Non-union	2
Delayed union	4
Implant failure	3

**Figure 2:**
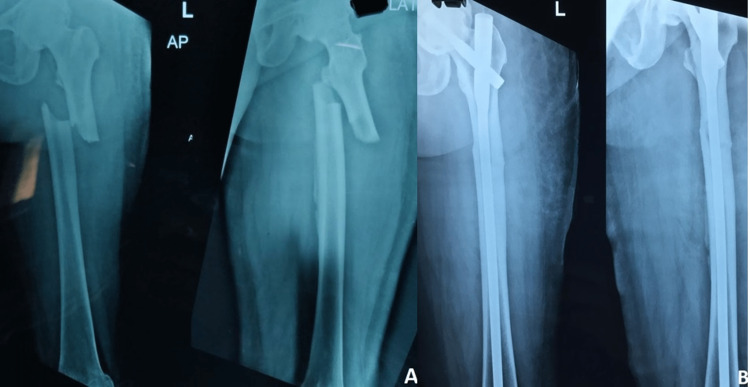
Pre-operative and one-year post-operative radiograph.

**Figure 3:**
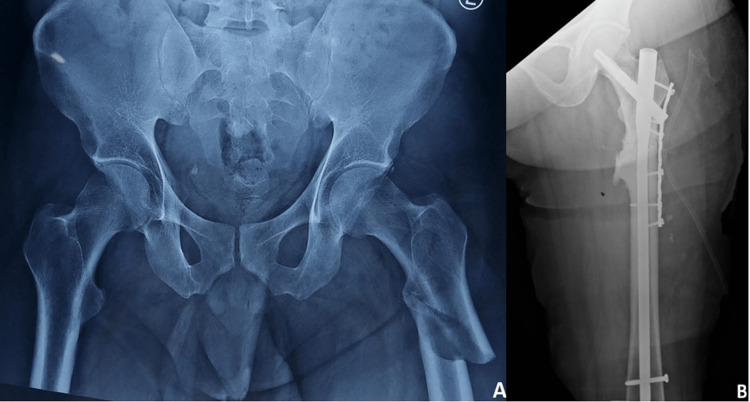
Pre-operative and one-year post-surgery radiograph.

The modified Hip Harris Score had a mean of 72.66 (Table 4) and 15 patients (about 75% of them) could walk normally again after a fracture. Representative pre-operative and one-year post-operative radiographs are presented in Figures 2-3.

**Table 4:** Modified Harris Hip Score. The data are displayed as the mean (median).

Modified Harris Hip Score	All (mean)
Post-operative three months	66.12
Post-operative six months	71.24
Post-operative one year	80.53

## Discussion

**Figure 4:**
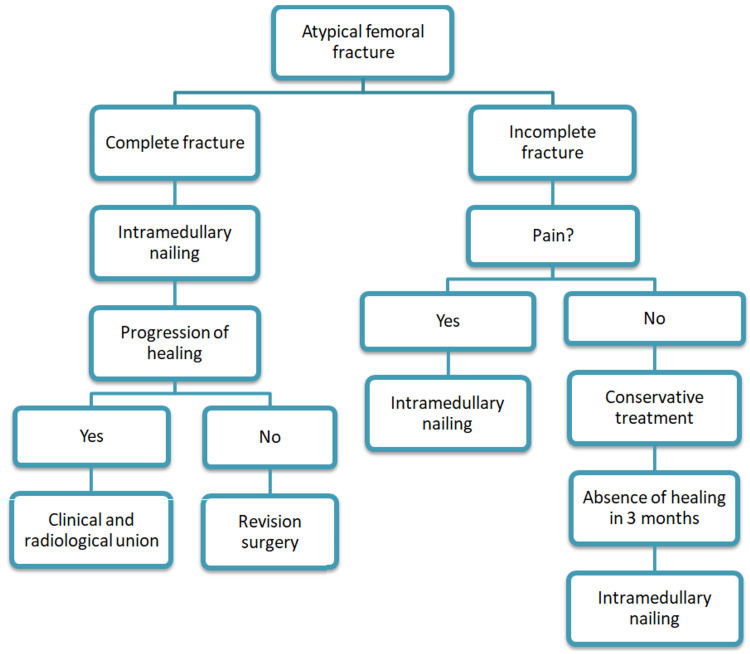
General treatment algorithm.

Most individuals with a history of stress fractures in the femur have used bisphosphonate drugs in the past. There is a growing concern that the use of bisphosphonates, a treatment for osteoporosis, may be contributing to an increase in atypical femur fractures among the elderly. Endochondral ossification, which includes the early stages of inflammation, callus deposition, and remodelling stages, is generally how complete fractures heal. Stress fractures, on the other hand, heal through bone remodelling. The use of bisphosphonates in patients should be taken into account when deciding on the surgical procedure for an atypical femoral fracture because they do not affect the early stages of inflammation and callus formation, but rather do so by preventing osteoclast recruitment and osteoclastic adhesion, limiting the life span of osteoclasts and suppressing the bone remodelling process [11,12]. Plate fixation has a high surgical failure rate due to the suppression of endochondral ossification, whereas nailing is associated with better outcomes [1,2]. A group of experts from the ASBMR concluded that full-length intramedullary nails are the preferred treatment option for atypical femoral fractures [3]. But the authors of this research have had new stress or secondary femoral neck fractures around the interlocking screw-nail interference after using a conventional interlocking intramedullary nail to treat atypical femoral fractures. This is why we think that using a cephalo-medullary nail is the best option [13]. Figure 4 illustrates the general treatment algorithm for an atypical femoral fracture.

A prospective cohort study was carried out by Ekstrom et al. [14], and the researchers discovered unfavourable results from the study. In terms of daily living activities, 50% of the patient population was unable to return to their pre-fracture level. The patient's two-year mortality was 25%.

Prasarn et al. did a retrospective study that compared fractures treated with and without bisphosphonates. They found that AFFs treated with bisphosphonates had a higher rate of postoperative plate failure (30%) than similar fractures not treated with bisphosphonates (0%) and also that there was no evidence of implant failure in AFFs that were treated with an intramedullary interlocking nail [15]. Fracture union times in their study were significantly higher than those seen in ordinary femur shaft fractures, averaging 26 weeks. Delays in healing were thought to be primarily caused by variations in bone quality. Teo et al. evaluated atypical subtrochanteric fractures in females and recorded the surgical outcomes for 33 patients in a single series. Implant failure and revision rates were 29.0% and 38.1%, respectively, for patients who had extramedullary implants, compared to 11.1% and 22.2% for patients who had intramedullary devices [16]. According to Ha et al., 10 atypical femoral fracture patients were documented in the study, and all of them healed between three and six months following surgery [17]. In a study by Lovy et al., 11 patients were given an injection of bone marrow aspirate and intramedullary fixation. The patients had good results and healed quickly [18]. Egol et al. treated 33 patients with 41 atypical femur fractures associated with at least five years of bisphosphonate use. They reported a high rate of union with intramedullary nailing; 98% of the fractures eventually fused at a mean time of 8.3 months [19]. Research on the usefulness of osteoinductive agents or autologous bone grafting in non-unions following treatment of atypical femoral neck fractures is lacking.

In our study, 11 atypical fractures, or 55%, had satisfactory bone union within six months, while four fractures, or 20%, were classified as having delayed union. Five patients experienced non-union with implant failure. There were a total of five revision procedures that included bone grafting, and the patient is still being evaluated and monitored in the outpatient department. In our group of patients, 15 (75%) were able to get back to the level of mobility they had before the fracture.

The retrospective study's design is simply one of the paper's flaws. This research will benefit orthopeadic surgeons who treat atypical femur fractures. However, prospective randomised trials with a larger sample size can provide a better understanding of this type of rare fracture.

## Conclusions

Atypical femur fractures are rare fractures that must be treated effectively with surgical fixation. Implant failure and non-union remain issues when treating these types of fractures. Successful treatment of AFF is possible by the use of intramedullary fixation, which enhances axial stability, serves as an internal splint, and lessens the likelihood of implant failure. A good functional and radiological prognosis can come from a stable fixation and a fracture that has been reduced anatomically.
